# ClinVar data parsing

**DOI:** 10.12688/wellcomeopenres.11640.1

**Published:** 2017-05-23

**Authors:** Xiaolei Zhang, Eric V. Minikel, Anne H. O'Donnell-Luria, Daniel G. MacArthur, James S. Ware, Ben Weisburd

**Affiliations:** 1 National Heart and Lung Institute, Imperial College London, London, SW7 2AZ, UK; 2 Royal Brompton Cardiovascular Research Centre, Royal Brompton & Harefield Hospitals NHS Trust, London, SW3 6NP, UK; 3 Analytic and Translational Genetics Unit, Massachusetts General Hospital, Boston, Massachusetts, 02114, USA; 4 Program in Medical and Population Genetics,, Broad Institute of MIT and Harvard, Cambridge, Massachusetts, 02142, USA; 5 Boston Children’s Hospital, Boston, Massachusetts, 02115, USA; 6 MRC London Institute of Medical Sciences, Imperial College London, London, W12 0NN, UK

**Keywords:** variant interpretation, ClinVar, XML parsing, Mendelian disease, pathogenic variants

## Abstract

This software repository provides a pipeline for converting raw ClinVar data files into analysis-friendly tab-delimited tables, and also provides these tables for the most recent ClinVar release. Separate tables are generated for genome builds GRCh37 and GRCh38 as well as for mono-allelic variants and complex multi-allelic variants. Additionally, the tables are augmented with allele frequencies from the ExAC and gnomAD datasets as these are often consulted when analyzing ClinVar variants. Overall, this work provides ClinVar data in a format that is easier to work with and can be directly loaded into a variety of popular analysis tools such as R, python pandas, and SQL databases.

## Introduction

ClinVar ^1^ is a public database hosted by the National Center for Biotechnology Information (NCBI) for the purpose of collecting information on genotype-phenotype relationships in the human genome. One common use case for ClinVar is as a catalog of genetic variants that have been reported to cause disease. When interpreting variants for clinical or research use, this data is commonly paired with reference data from ExAC (Exome Aggregation Consortium) or gnomAD (Genome Aggregation Database) ^2^, particularly for large-scale analyses of reportedly pathogenic variants.

ClinVar makes its data available via FTP in three formats: XML, TXT, and VCF. However, none of these files were ideally suited for large scale data collection and downstream analysis for variant interpretation. The VCF file only contains variants present in dbSNP, which is not a comprehensive catalog of ClinVar variants. The TXT file is organized around Allele ID and the reported clinical significance is aggregated over distinct disorders. It also lacks certain annotations such as PubMed IDs for related publications, inheritance mode, prevalence, and other disease related information. The XML file is a comprehensive representation, but is organized around unique variant-condition combinations and is large and complex, making it difficult to quickly look up a variant of interest, and many potential users considering larger scale analyses may not be familiar with tools required to parse this data format. In addition, both the XML and TXT representations contain many genomic coordinates that have been parsed from HGVS notation. Therefore these representations may be right-aligned in contrast to VCF standard ( http://samtools.github.io/hts-specs/VCFv4.1.pdf), and may also be non-minimal, i.e. containing additional nucleotides of context to the left or right of a given variant ^3^.

To facilitate access to accurate and comprehensive ClinVar data at scale, we developed this software tool to convert the latest raw ClinVar data into multiple data files with options specified by users. The tool supports both genome build GRCh37 and GRCh38. For each genome build, ClinVar records are parsed into separate files for simple mono-allelic variants and complex variants (i.e. with more than one variant interpreted together such as compound heterozygous and haplotype), and are further separated by whether variant records are variant-condition specific or aggregated for distinct conditions. The variant records are also annotated with reference population data from ExAC or gnomAD. The resulting files provide a summary of the most relevant fields for a range of uses in an accessible format.

## Methods

### Implementation

**Figure 1.**
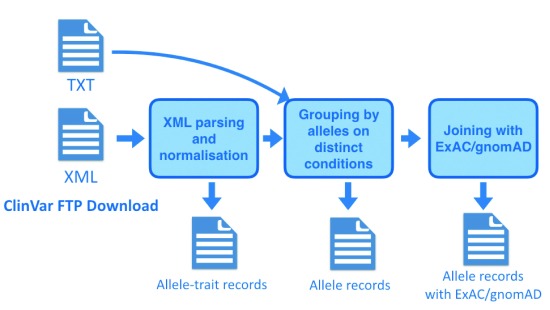
The workflow to parse ClinVar data.

To create a flat representation of ClinVar data suited for general purposes, we took several steps illustrated in Figure 1, which are encapsulated in the pipeline src/master.py:

- Download the latest XML and TXT files from ClinVar FTP.
- Parse the XML file to extract fields of interest into a flat file.
- Normalize the representation of genome coordinates using our Python implementation of vt normalize ^3^.
- Group the allele-trait records by allele to aggregate record information except clinical significance from multiple submitters, independent of conditions. Join the TXT file to aggregate the clinical significances from multiple submitters and generate VCF files.
- Join with ExAC or gnomAD data and generate table files.

In the parsing process, when grouping the same allele over distinct conditions, we defined additional columns to indicate the clinical significance, since one allele may contain multiple assertions of clinical significance:

- Pathogenic is 1 if the variant has ever been asserted as "Pathogenic" or "Likely pathogenic" by any submitter for any phenotype, and 0 otherwise
- Benign is 1 if the variant has ever been asserted as "Benign" or "Likely benign" by any submitter for any phenotype, and 0 otherwise
- Conflicted is 1 if the variant has ever been asserted as "Pathogenic" or "Likely pathogenic" by any submitter for any phenotype, and has also been asserted as "Benign" or "Likely benign" by any submitter for any phenotype, and 0 otherwise. A variant having one assertion of pathogenic and one of uncertain significance does not count as conflicted for this column.

### Operation

The pipeline is written in python 2.7 and R. It depends on python packages pysam (v0.11.1), pandas (v0.19.2) and pypez (v0.1.5). Additionally, the tabix ^4^ and vt ^3^ third-party tools must be installed and executable from any directory.

To run the pipeline:

                        
cd ./src
pip install –user –upgrade -r requirements.txt
python2.7 master.py –b37-genome /path/to/b37.fa –b38-genome /path/to/b38.fa \
-E /path/to/ExAC.vcf.gz -GG /path/to/gnomad.genomes.vcf.gz -GE /path/to/gnomad.exomes.vcf.gz
                    


## Use case

Whiffin *et al.* (2016) ^5^ made use of the tool to efficiently extract the interpreted variants for inherited cardiovascular diseases from ClinVar, and used the variant allele frequency from ExAC to reassess the variant pathogenicity.

## Limitations

The accuracy of output files is limited by the downloadable files from the ClinVar FTP site. In the case that ClinVar releases new data with a new reporting format or an unfinished format update, our pipeline may not work for the latest release. We would recommend that users revert to the old version by specifying the input ClinVar files when executing python master.py.

## Summary

The software tool is developed to parse the latest ClinVar release data into analysis-friendly files to facilitate convenient data collection in variant interpretation research. The files are separated by genome build (GRCh37/GRCh38), and by whether they represent simple mono-allelic variants, or complex multi-allelic variants such as compound-heterozygotes and haplotypes. Records are annotated with reference population data from ExAC and/or gnomAD. The representation of variant genome coordinates are normalized and the resulting output files are suitable for multi-purpose downstream variant-interpretation analysis.

## Software availability

The pipeline, its fully parsed data files and example data files are available: https://github.com/macarthur-lab/clinvar

Archived source code as at the time of publication: https://doi.org/10.5281/zenodo.399052

License: The software tool is distributed under an MIT license. ClinVar data, as a work of the United States federal government, are in the public domain and are redistributed here under the same terms as they are distributed by ClinVar itself. Importantly, note that ClinVar data are "not intended for direct diagnostic use or medical decision-making without review by a genetics professional".
