# Supplementary material for: ClinVar data parsing
Source: Wellcome Open Res. 2017 May 23;2:33. [Version 1] doi: 10.12688/wellcomeopenres.11640.1 (PMC5473414; doi:10.12688/wellcomeopenres.11640.1)
Supplement: Peer review history

# Peer review: Referee response for version 1

**DOI:** 10.21956/wellcomeopenres.12572.r22989

**Published:** 2017-06-12

**Authors:** Steven M Harrison

**Affiliations:** 1 Laboratory for Molecular Medicine, Harvard Medical School, Boston, MA, USA

This paper by Zhang *et al* provides a mechanism to combine ClinVar data downloads into a format that is easier to work with and implement, as ClinVar downloads are either not fully comprehensive individually or too large and complex for the average ClinVar user to use. By including variant frequency information from gnomAD/ExAC, the authors have created a valuable resource for the genetics community. This paper is incredibly timely as more clinical laboratories and researchers utilize ClinVar and population frequency data, but incorrect implementation of data from either source can lead to incorrect filtration and classification.

 To help user better understand and implement this resource, it may be helpful to change the logic for "conflicted" to instead indicate all medically significant conflicts (pathogenic/likely pathogenic versus uncertain significance/likely benign/benign) as opposed to just pathogenic/likely pathogenic versus likely benign/benign. For example, ClinVar variant https://www.ncbi.nlm.nih.gov/clinvar/variation/186876/ has 6 submissions: 1 of Likely pathogenic and 5 of Uncertain significance. To mark the variant as "1" for "pathogenic" without an indication in the "conflicted" column could be misleading as it's not clear that the majority of submitters actually think this variant is of uncertain significance. Alternatively listing the clinical significance terms with counts would help, such as "Likely pathogenic(1);Uncertain significance(5)" but perhaps that is not available until ClinVar moves to a variant-centric XML download.

 Additionally, it may be helpful to move from RCV#s to SCV#s when representing ClinVar records. Users who navigate to an RCV page may not be aware that they are only looking at interpretations regarding a variant/condition pair and not seeing all interpretations submitted for the variant. Also, by listing all SCV#s, it would be clearer to users how many total submissions there are for any given variant. Clarifying that measureset_id = VariationID could also be helpful as many ClinVar users are familiar with the VariationID concept but likely unaware of its name in the XML.

 Lastly, if possible, it would be incredibly helpful if coverage data from gnomAD/ExAC could be added for variants in ClinVar that are absent from gnomAD/ExAC to help users determine the difference between absent due to poor coverage versus truly absent from tested alleles.

I have read this submission. I believe that I have an appropriate level of expertise to confirm that it is of an acceptable scientific standard.

# Peer review: Referee response for version 1

**DOI:** 10.21956/wellcomeopenres.12572.r22988

**Published:** 2017-06-05

**Authors:** Fiona Cunningham, Sarah Hunt

**Affiliations:** 1 European Molecular Biology Laboratory, Wellcome Trust Genome Campus, European Bioinformatics Institute, Cambridge, UK; 2 European Molecular Biology Laboratory, European Bioinformatics Institute, Cambridge, UK

The authors provide a tool to reformat a ClinVar data release into a set of simply formatted files. They augment the ClinVar information with frequency data from the ExAC and gnomAD projects, which are the popular reference sets used in the community.

 Existing tools like VEP and SNPeff allow variants to be annotated with allele frequencies from popular reference sets and ClinVar clinical significance classifications. They provide some but not full phenotype and evidence details.

 ClinVar provides data in multiple file formats which are of different degrees of readability and parse-ability and contain different data types. Extracting the full information from these files can be non trivial, so this tool will be of use to groups wishing to use ClinVar data in large scale analyses.

 The normalization step is essential to avoid loosing potential matching records, but it would add clarity to state this step left-aligns allele representations as some groups use a different convention. Some information on the performance of the pipeline would also be useful.

 All in all though, this is a useful tool for the community.

We have read this submission. We believe that we have an appropriate level of expertise to confirm that it is of an acceptable scientific standard.
